# Supplementary material for: Hydrogen-Rich Water Mitigates LPS-Induced Chronic Intestinal Inflammatory Response in Rats via Nrf-2 and NF-κB Signaling Pathways
Source: Vet Sci. 2022 Nov 8;9(11):621. doi: 10.3390/vetsci9110621 (PMC9692594; doi:10.3390/vetsci9110621)

veterinary sciences

File S1: Original images of Western Blotting

TLR4

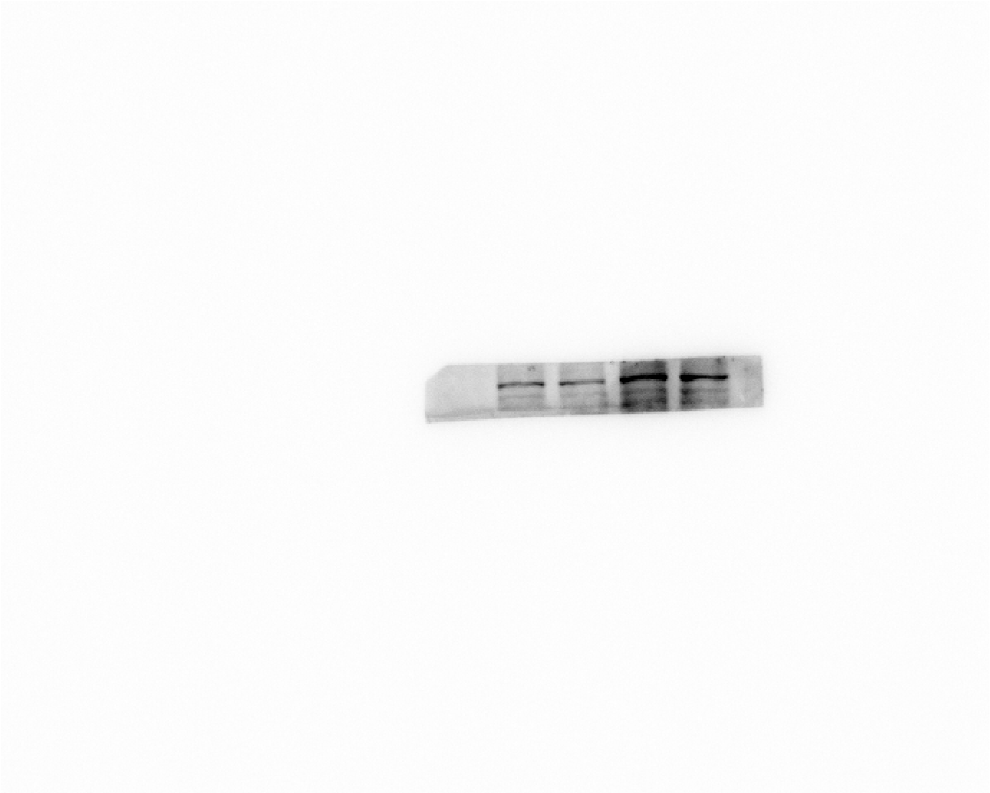

p65

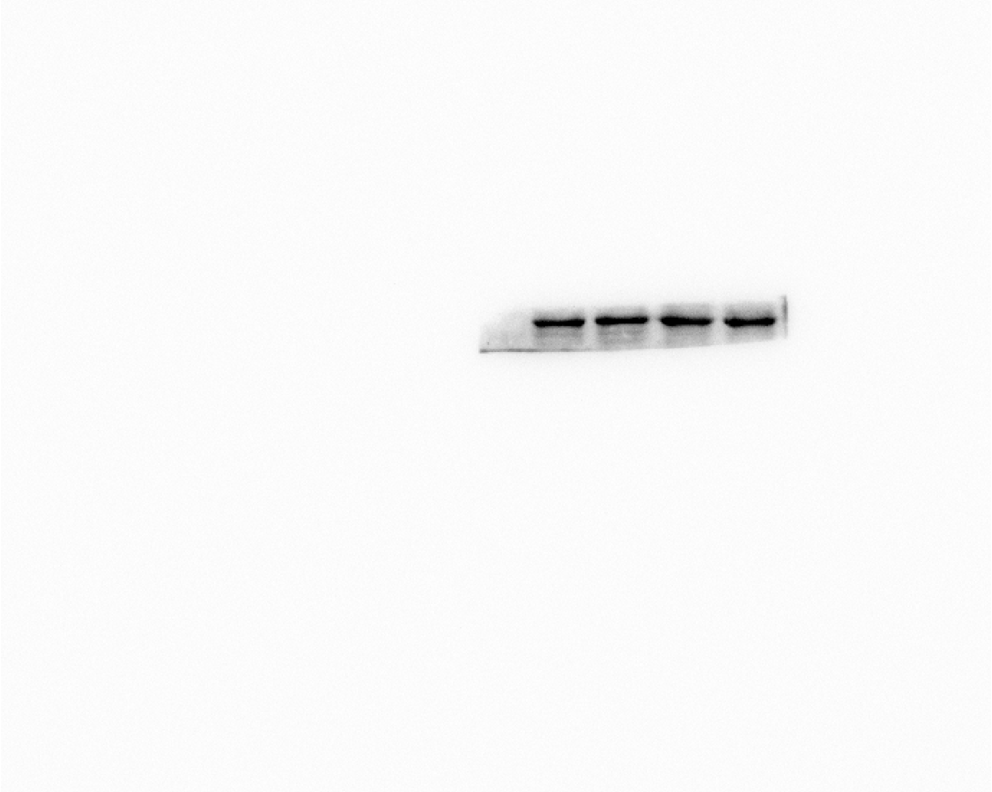

p-p65

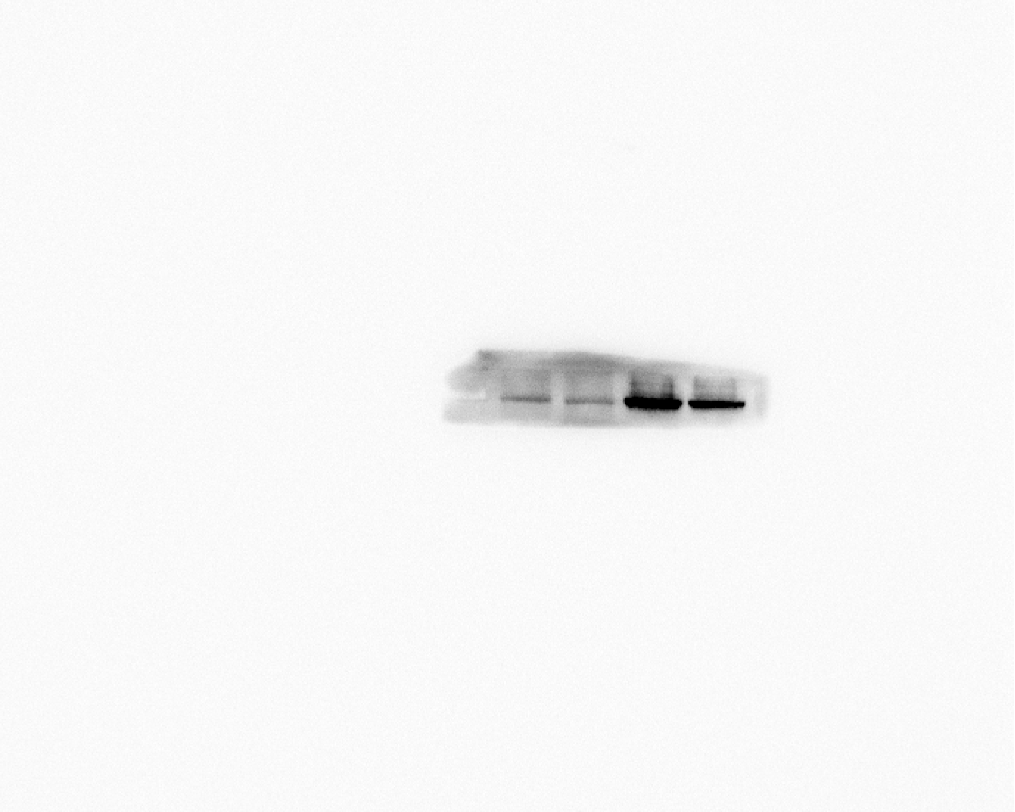

P50

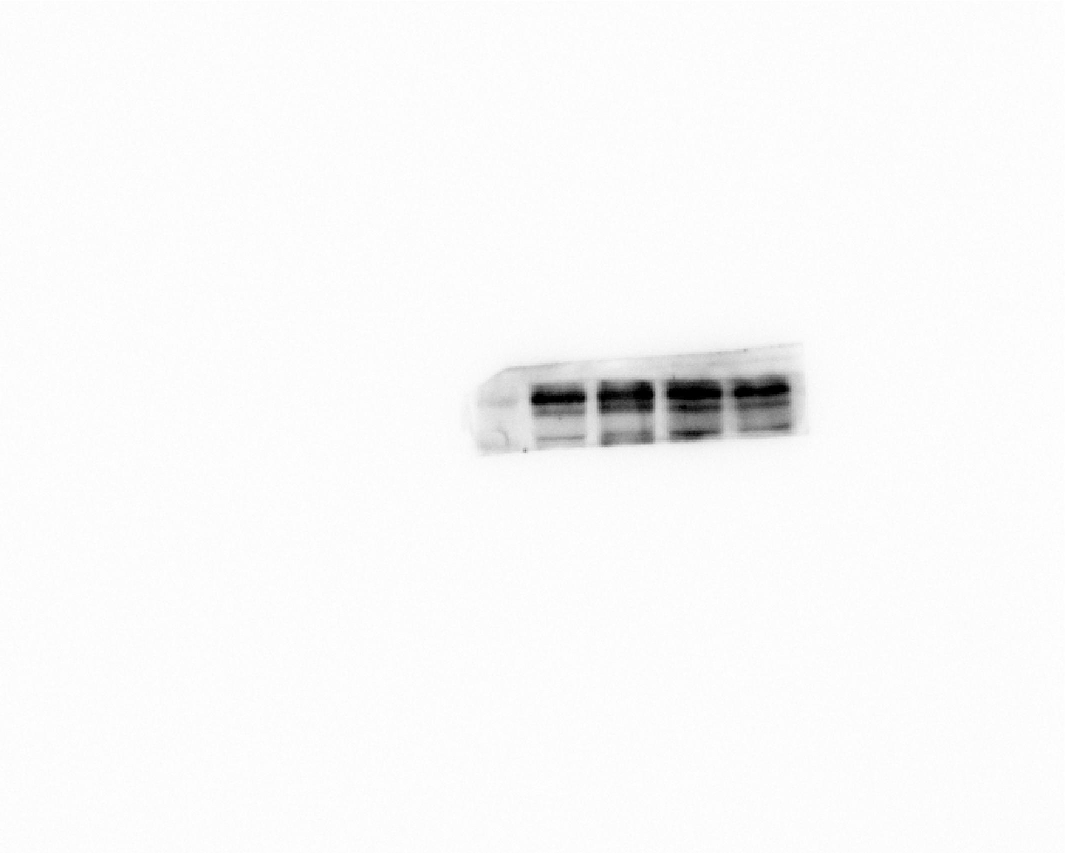

p-p50

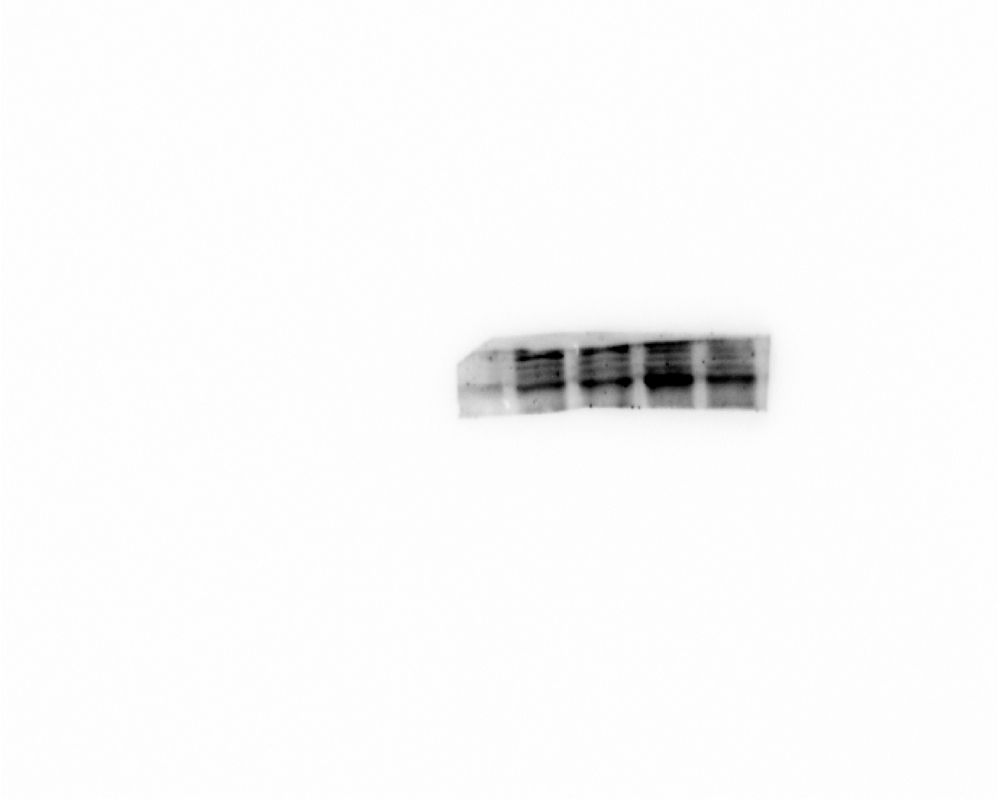

MyD88

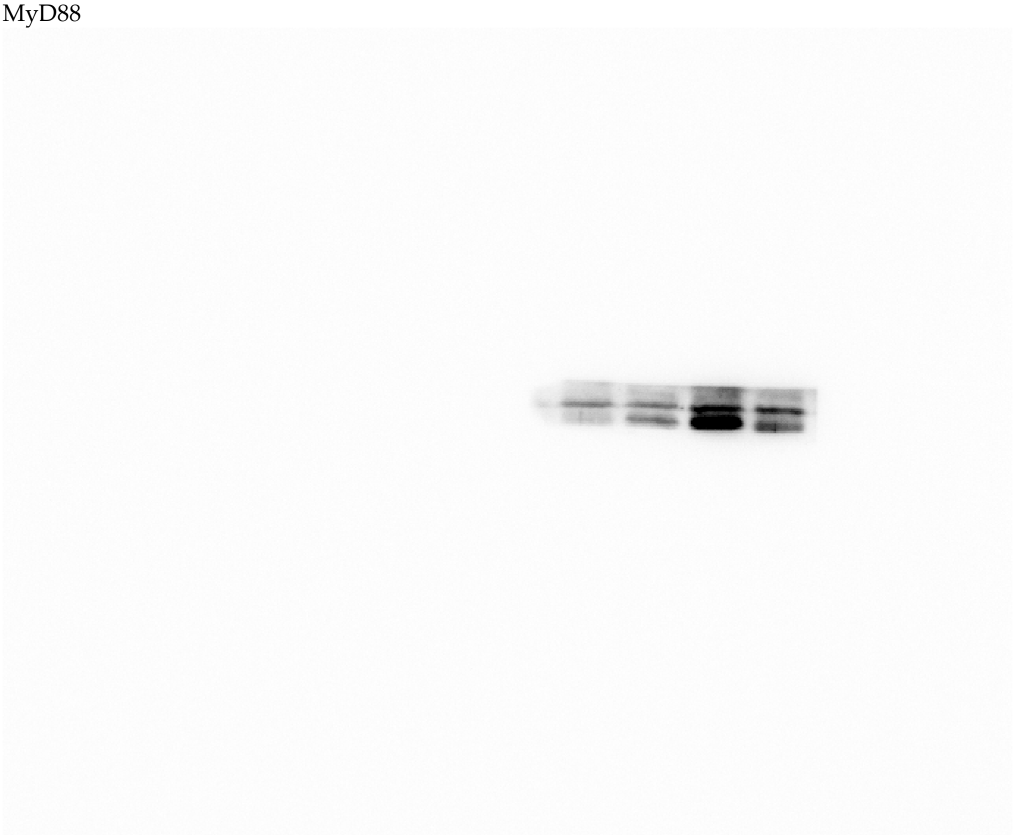

IκBα

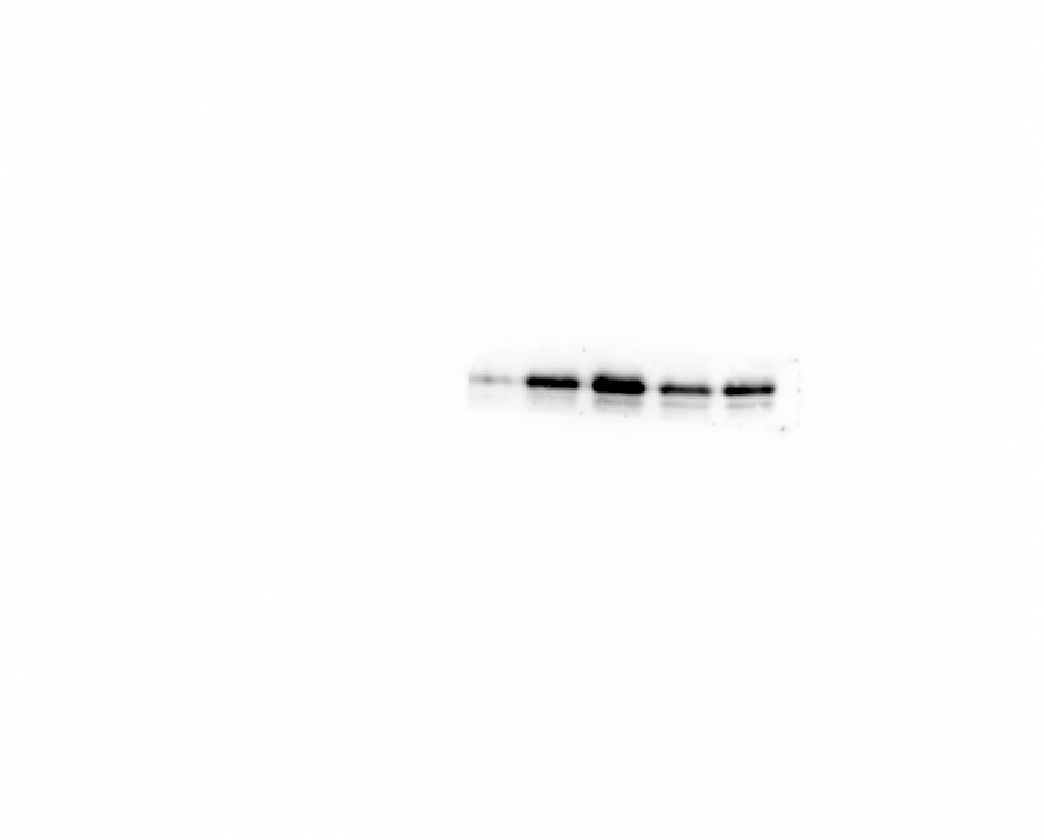

p- IκBα

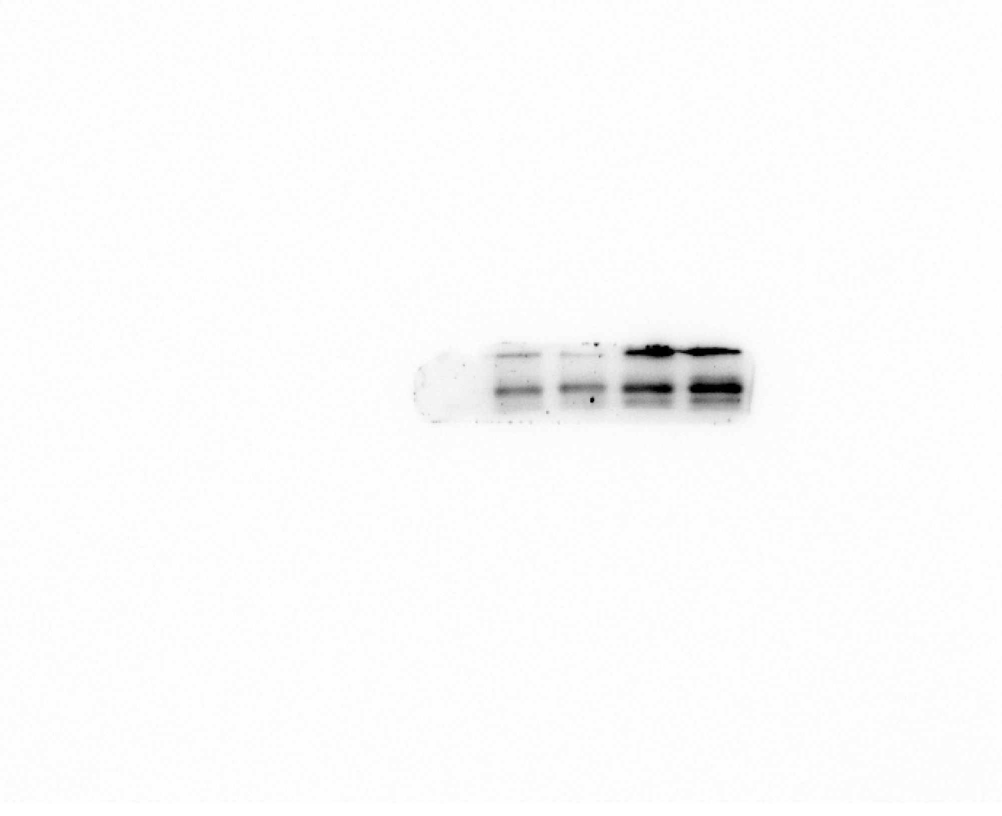

Actin

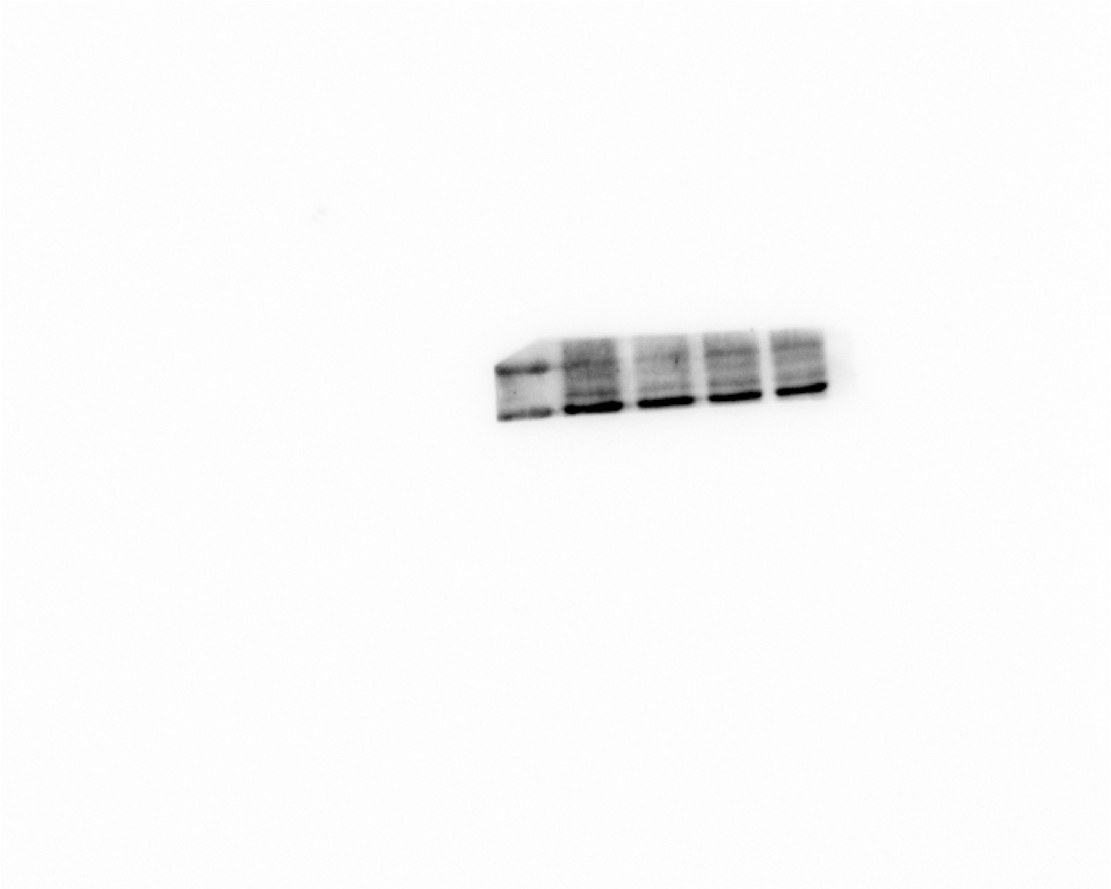

Nrf-2

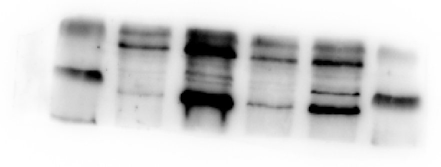

HO-1

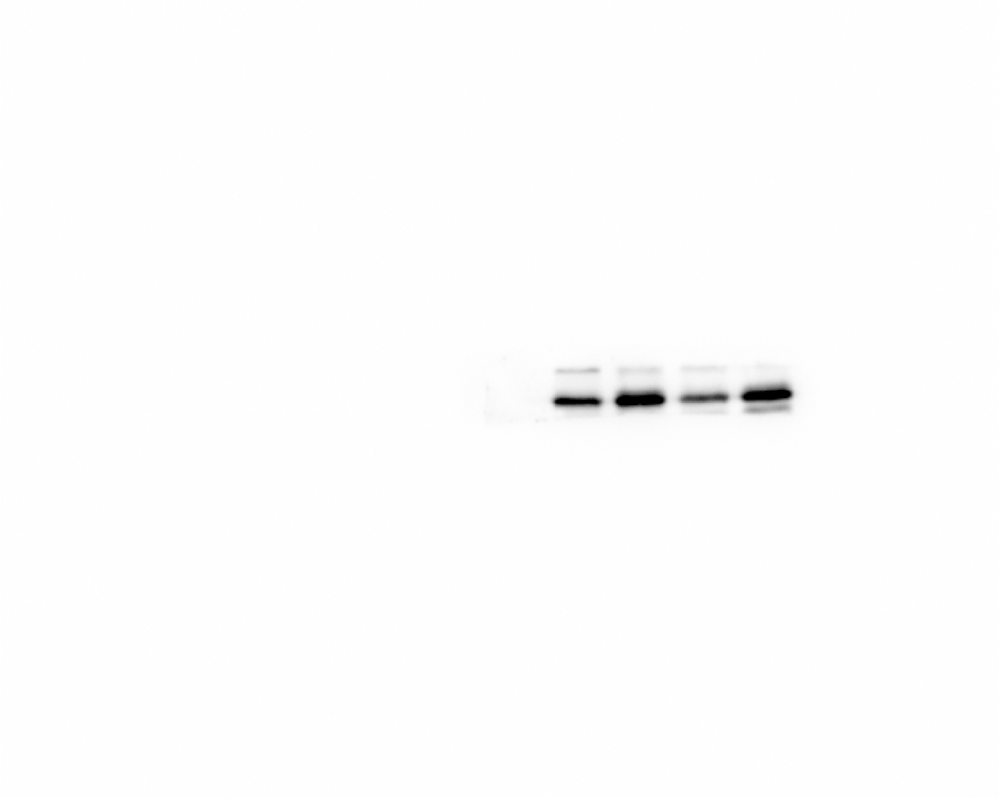

NQO-1

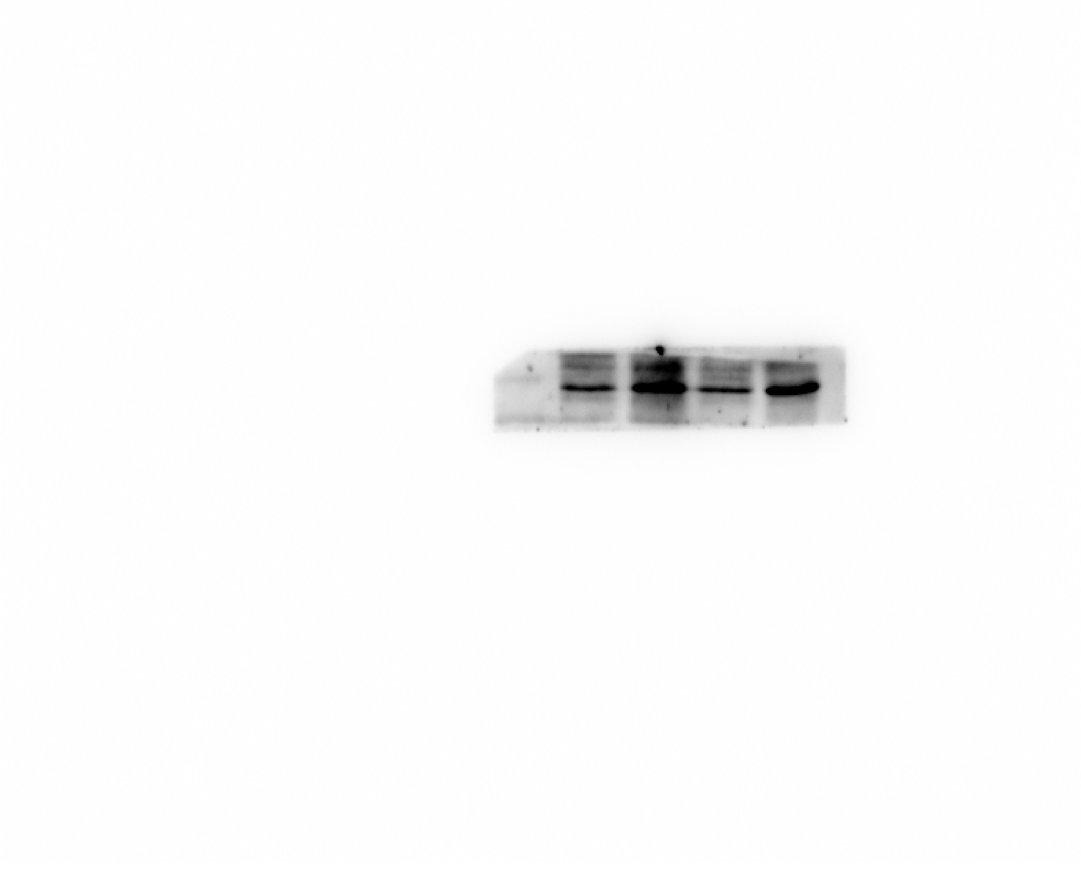

Actin

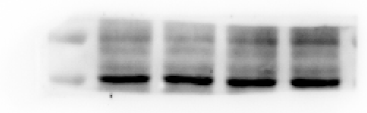

Supplement: Supplementary file 1 [file vetsci-09-00621-s001.zip › vetsci-1940128-Supplementary.pdf]
